# A 500-m Agricultural Drought Impact Dataset in China’s Main Grain Region: Toward Impact-Based Drought Monitoring

**DOI:** 10.1038/s41597-026-06732-3

**Published:** 2026-02-05

**Authors:** Jiali Shi, Yan-Fang Sang, Amir AghaKouchak, Sonam Sandeep Dash, Faith Ka Shun Chan

**Affiliations:** 1 Key Laboratory of Water Cycle & Related Land Surface Processes, Institute of Geographic Sciences and Natural Resources Research, Chinese Academy of Sciences, Beijing, 100101 China; 2 University of Chinese Academy of Sciences, Beijing, 101407 China; 3 Key Laboratory of Compound and Chained Natural Hazards, Ministry of Emergency Management of China, Beijing, 100085 China; 4 Department of Civil and Environmental Engineering, University of California Irvine, CA 92697 Irvine, USA; 5 United Nations University Institute for Water, Environment and Health (UNU-INWEH), Ontario, Canada; 6 Physical Science and Engineering Division, King Abdullah University of Science and Technology, Thuwal, Saudi Arabia; 7 School of Geographical Sciences, Faculty of Science and Engineering, University of Nottingham Ningbo China, Ningbo, 315100 China

**Keywords:** Hydrology, Natural hazards, Climate sciences

## Abstract

A high-resolution, quantitative dataset of agricultural drought impacts is essential for advancing impact-based drought monitoring and prediction. Yet, such data remain the critical missing piece, representing the major obstacle to developing robust, impact-driven drought assessments. Here, we generated a 500 m-gridded agricultural drought-impacted area dataset in the China’s main grain region (ADIA-CMGR) during 2006–2020. We employ a leaf area index (LAI)-based relative threshold method to extract the areas with three degrees of drought impacts for summer-harvest crops, autumn-harvest crops, and early rice, respectively. The dataset constitutes various information, including *drought-covered area*, *drought-damaged area*, and *crop failure area*. Validation with the text-based qualitative records of agricultural drought-impacted areas shows that ADIA-CMGR offers accurate temporal variability and reasonable spatial distribution. The developed dataset satisfactorily revealed the spatial and inter-annual dynamics of agricultural drought-impacted areas across various crop-growing seasons, providing a solid foundation for managing drought impacts and improving agricultural practices.

## Background & Summary

Unlike sudden-onset hazards, droughts evolve slowly yet exert profound and lasting impacts on ecosystems, agriculture, and water resources around the globe^1,2^. Drought adversely impact the terrestrial ecosystem and human society, with 82% of drought impacts prevalent in the agricultural sector^3,4^. For instance, agricultural droughts have reduced global crop yield by nearly 10% during the past five decades^5^. These plausible damages are expected to be aggravated further in the context of future climate change and variability^6^. The Global Drought Snapshot reported by the 28th United Nations Climate Change Conference (COP28) in 2023 highlighted the importance of taking proactive actions to better assess the drought-related impacts on agriculture and allied sectors^7^.

In recent years, impact-based agricultural drought monitoring has gained urgency, highlighting the need for improved strategies to mitigate drought-related losses and support sustainable agricultural development^8,9^. Several studies adopted multiple drought indices to assess and predict agricultural drought conditions, and subsequently linked them to specific agricultural drought impacts (e.g. yield reduction and crop failure caused by droughts)^10–14^. Certainly, these outcomes can quantify the severity, magnitude and damages of specific agricultural drought impacts, enabling water managers, stakeholders, and policy makers to better prepare for the timely adoption of preventive measures.

Agricultural drought impact data provide useful insights on the actual losses and their spatiotemporal variation, and they are the backbone of impact-based agricultural drought assessment. At present, current agricultural drought impact data can be categorized into two groups: text-based reports and statistical surveys^15^. Text-based reports systematically aggregate the information of actual agricultural drought impacts collected from newspapers, online media, government reports, and expert surveys. Few databases (e.g. US Drought Impact Reporter; European Drought Impact Report Inventory) were developed by primarily integrating and reclassifying text-based reports^16,17^. However, most text-based reports just provided qualitative or semi-quantitative information about actual drought impacts, which cannot meet the need of a quantitative assessment of agricultural drought impacts^15^. In contrast, statistical surveys provide quantitative impacts of agricultural droughts, such as annual crop yield reduction and agricultural drought-affected area, while they are commonly recorded by the local administrative entities, and are mostly confined to annual-scale and county- or provincial-scales in the public domain^18^. However, these statistical surveys cannot capture detailed information on specific impacts of different crops and impacts across different jurisdictions, limiting their applicability in fine-resolution agricultural drought research. Therefore, a high-resolution quantitative dataset of agricultural drought impacts has been recognised as “the missing piece” in impact-based agricultural drought research^15,19^.

Under such sparse scenarios of drought impact data, the remote sensing-based agricultural drought impact assessment is the most feasible alternative for generating continuous, high-resolution, and quantitative agricultural drought impact dataset. Past studies applied two main remote sensing-aided approaches to assess agricultural drought impacts. The first approach assimilates remote sensing data into crop models to enhance the spatial simulation capability of crop growth or crop yield^20,21^. The second relies on drought indices which are derived from different spectral bands to characterize crop growth, and then links these drought indices to actual drought impacts or yield loss using suitable statistical methods^22^. The first approach is typically used in the drought impact assessment for one or a few crop types. In contrast, the latter is more flexible in large-scale agricultural drought impact assessment. Therefore, in this study we apply the second approach to evaluate agricultural drought impacts by establishing the statistical relationship between drought indices and actual drought impacts. Among all drought indices, the vegetation indices have been proven to be the most direct driver for understanding actual crop growth conditions and, consequently, yield outcomes^23–25^. Among them, the leaf area index (LAI) can reflect the amount of photosynthesis and dry matter accumulation during the reproductive stage, and it performs much better than other vegetation indices like Normalized Difference Vegetation Index, the Vegetation Condition Index, and the Vegetation Health Index, signifying its ability to reveal the agricultural drought impacts on crops^26^.

China is one of the most important grain-producing countries in the world, feeding nearly 19% of the world’s population with availability of only 7% of the global arable land^27^. Despite its dependency on agriculture, China has been seriously affected by agricultural droughts. The mean annual agricultural drought-affected area in China has reached 20 million hectares over the last few decades^28^. Hence, this study focused on the China’s main grain region (CMGR) and aimed to generate a high-resolution agricultural drought-impacted area dataset encompassing the CMGR. The moderate resolution imaging spectroradiometer (MODIS) satellite-derived LAI product, as well as the historical drought statistics of agricultural drought areas from the Bulletin of Flood and Drought Disasters in China (BFDDC)^29^, are used for this research. We employ the LAI-based relative threshold method developed in a previous study^26^ to extract the areas with three degrees of drought impacts for summer-harvest crops, autumn-harvest crops, and early rice, respectively. The three degrees correspond to drought-covered area (sown area with drought-caused yield losses >10%), drought-damaged area (sown area with drought-caused yield losses >30%), and crop failure area (sown area with drought-caused yield losses >80%). Subsequently, the dataset of 500 m-resolution agricultural drought-impacted areas over the CMGR, called ADIA-CMGR, was generated, spanning the period of 2006–2020. The accuracy of ADIA-CMGR was evaluated using the historical records of agricultural drought impacts in BFDDC and other literature. This new dataset holds significant potential in improving drought response and agricultural practices, and optimising the agricultural water management at fine spatial scales across China.

## Methods

### Study area

**Fig. 1:**
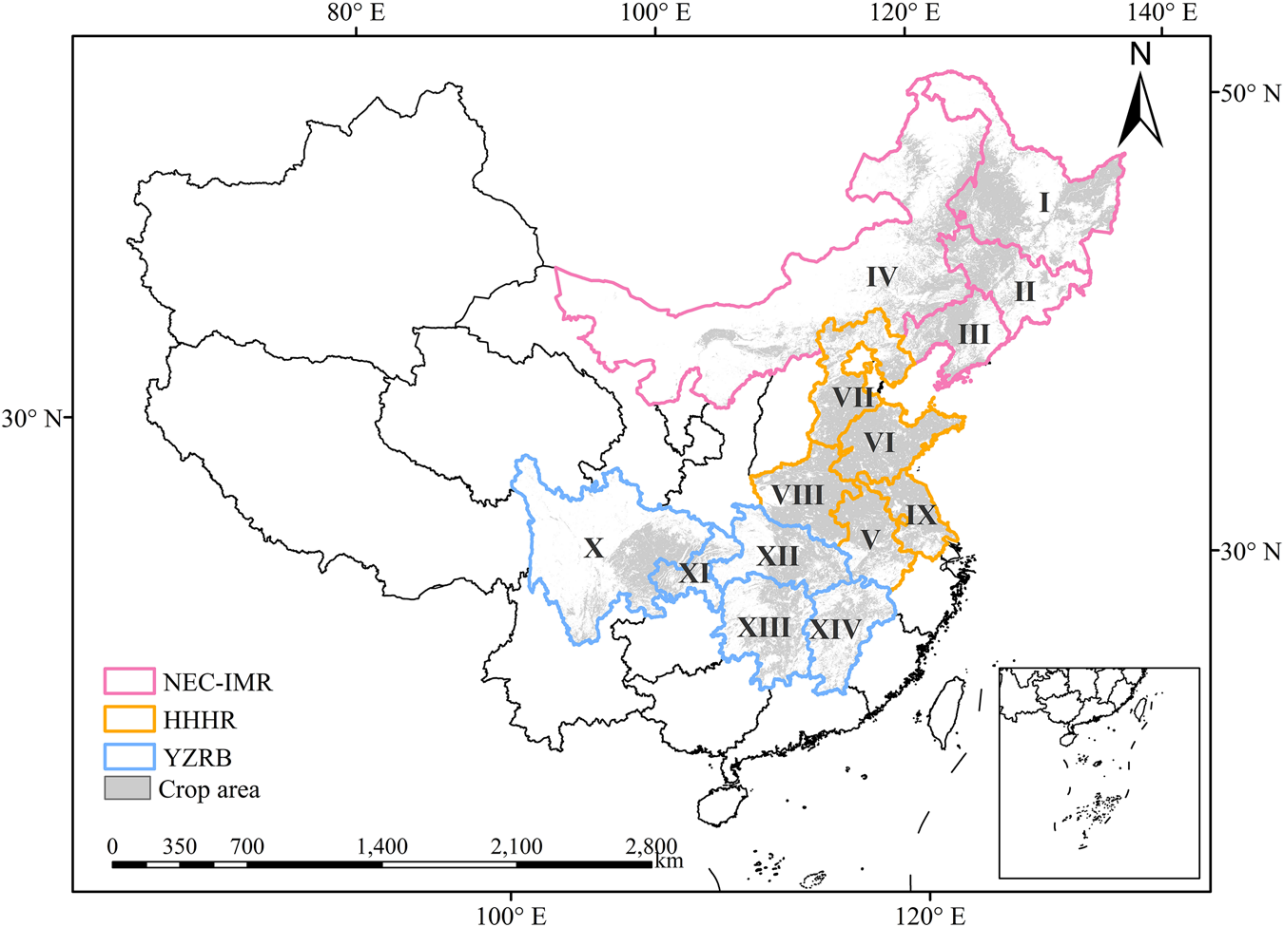
Locations of CMGR, including the Northeast China and Inner Mongolia region (NEC-IMR), the Huang-Huai-Hai region (HHHR), and the Yangtze River Basin (YZRB); and the spatial distribution of crop area concerned in this study. I: Heilongjiang; II: Jilin; III: Liaoning; IV: Inner Mongolia; V: Anhui; VI: Shandong; VII: Hebei; VIII: Henan; IX: Jiangsu; X: Sichuan; XI: Chongqing; XII: Hubei; XIII: Hunan; XIV: Jiangxi.

The CMGR accounts for 75% of China’s sown area and contributes to approximately 79% of China’s total grain production (Fig. 1)^30^. This region covers 13 provinces, including Heilongjiang, Jilin, Liaoning, Inner Mongolia, Anhui, Shandong, Hebei, Henan, Jiangsu, Sichuan, Hubei, Hunan, and Jiangxi. To keep the spatial integrity of the study area, Chongqing was also included in this research (Fig. 1). The study area is characterised by a complex environment and diverse crop planting structures (Tab. S1). Based on crop growing seasons and crop phenological calendars (see Table S1), we divided the study area into three sub-regions: the Northeast China and Inner Mongolia region (NEC-IMR), the Huang-Huai-Hai region (HHHR), and the Yangtze River Basin (YZRB) (Fig. 1). The three sub-regions have different growing seasons, each of which corresponds to a specific drought-impacted area map for every year, and the sum of them constitutes the yearly agricultural drought-impacted areas. The NEC-IMR has only one growing season, with one agricultural drought-impacted area map for autumn-harvest crops available annually. The HHHR has two main growing seasons, producing two agricultural drought-impacted area maps for summer-harvest crops and autumn-harvest crops, respectively in each year. In Hunan and Jiangxi of YZRB, they have a specific growing season of early rice and thus have three agricultural drought-impacted area maps for summer-harvest crops, autumn-harvest crops, and early rice, respectively. The rest three provinces (i.e., Sichuan, Chongqing and Hubei) of YZRB have two agricultural drought-impacted area maps for summer-harvest crops and autumn-harvest crops annually.

### Data collection and pre-processing

The data used in this study are primarily categorised into three components: historical agricultural drought information, crop information, and LAI data. Historical agricultural drought information of CMGR during 2006–2020 was acquired from the BFDDC published by the Ministry of Water Resources of the People’s Republic of China (http://mwr.gov.cn/sj/tjgb/zgshzhgb/). The BFDDC provides yearly agricultural drought-impacted areas of each province under three heads: drought-covered area, drought-damaged area, and crop failure area, representing the sown area with yield losses due to agricultural drought greater than 10%, 30%, and 80%, respectively. The BFDDC also provides detailed text-based reports of the spatial distribution of agricultural droughts, serving as important information for validating ADIA-CMGR generated in this study. This dataset has been widely used in analysing the spatiotemporal dynamics of agricultural drought, providing a reliable reference for the generation of a high-resolution agricultural drought area dataset^30,31^.

Crop information consists of crop coverage data and crop phenological calendar of the respective region. Crop coverage data include summer-harvest area, autumn-harvest area, and early rice area. Summer-harvest area and autumn-harvest area are obtained from the 500 m annual cropping intensity dataset for monsoon Asia (ACIA500)^32^. The summer-harvest area represents regions with double cropping, while the autumn-harvest area includes both single and double cropping regions. Specifically, in Hunan and Jiangxi of YZRB, the early rice area was acquired from the dataset of the harvest area for three staple crops in China, called as China Crop Area 1 km Dataset^33^. For the year 2020, the early rice area data was extrapolated based on the 2019 dataset. The summer-harvest area was calculated as the total double-cropping area, excluding the early rice area.

For this study, LAI data were acquired from the MODIS satellite-derived LAI product (MOD15A2H) (https://ladsweb.modaps.eosdis.nasa.gov/) with a spatial resolution of 500 m and a temporal resolution of 8 days^34^, which has demonstrated satisfactory performance in assessing the regional agricultural drought condition monitoring^35^. The LAI data was acquired using the Google Earth Engine platform. Moreover, the Savitsky-Golay filter tool in ENVI was applied to reduce noise of the MODIS LAI data^36^; the window size was set to 3 and the polynomial order was set to 3 for better capturing of change regimes for LAI.

All the above raster datasets were re-projected to the same coordinate reference system and resampled to 500 m resolution using the nearest-neighbour resampling approach by ArcGIS software.

### Determination of LAI thresholds

**Fig. 2:**
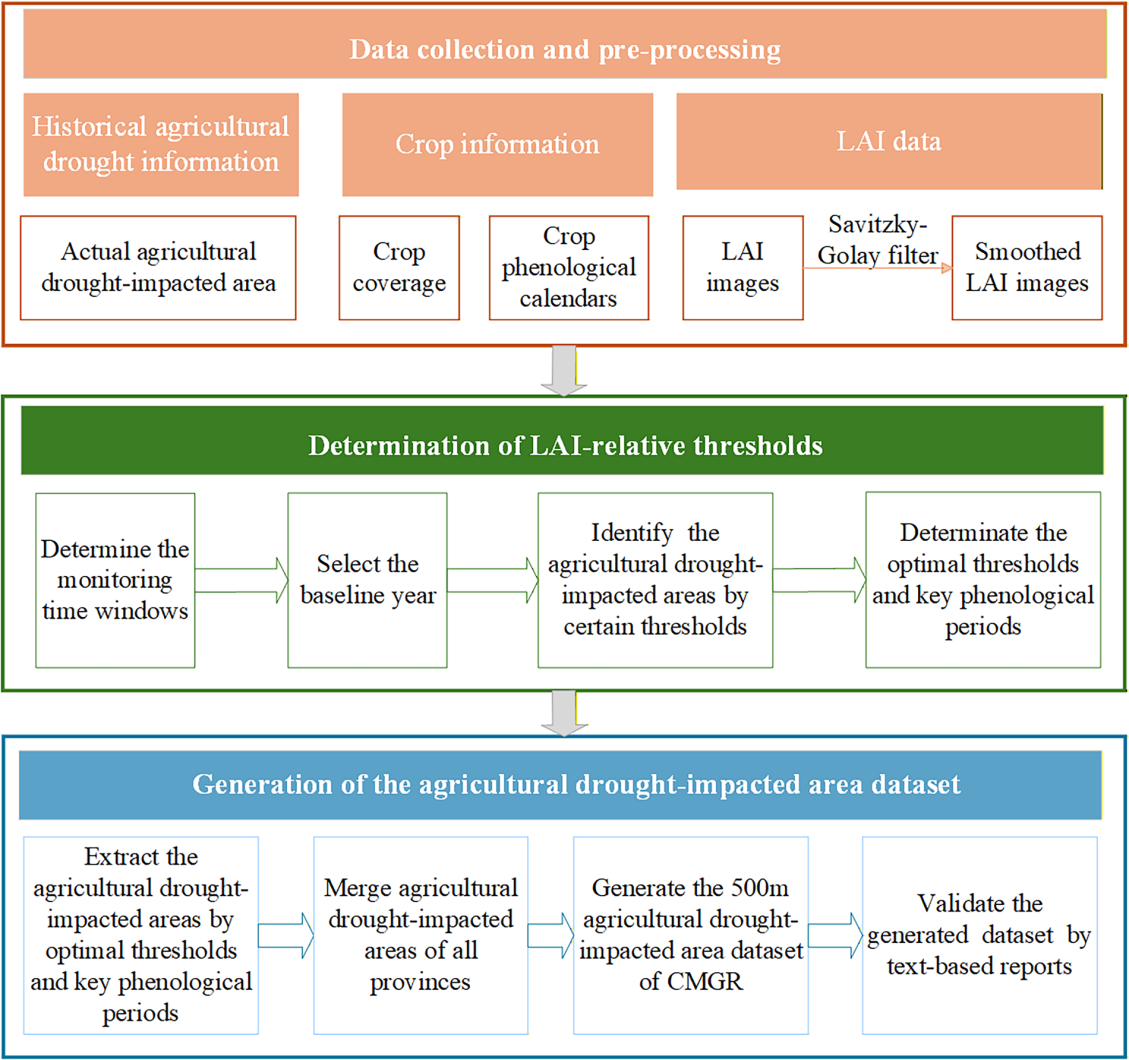
Workflow of generating the ADIA-CMGR dataset in this study.

We generated historical agricultural drought information, crop information, and LAI data to develop ADIA-CMGR. The overall methodological framework is shown in Fig. 2. A LAI-based relative threshold method^26^, was employed to determine the thresholds corresponding to drought-covered, drought-damaged, and crop failure areas for summer-harvest crops, autumn-harvest crops, and early rice in each province. Subsequently, the final ADIA-CMGR was generated by integrating agricultural drought-impacted areas across all provinces. To ensure the accuracy of ADIA-CMGR, we independently validated it using qualitative reports of agricultural drought impacts from the BFDDC and other relevant literature sources.

Given the significant spatial heterogeneity of LAI values arising from topographic features and crop physiological characteristics, the relative LAI thresholds were applied to quantify the different severity degrees of agricultural droughts. This approach helps avoid misclassifying regions with naturally low LAI values as persistently drought-impacted areas. The relative LAI thresholds represent the maximum percentages of the actual LAI relative to the baseline LAI in the same period when the yield loss is triggered to a certain degree. In line with the classification of drought impacts provided in the BFDDC —namely, drought-covered area, drought-damaged area, and crop failure area— the relative LAI thresholds in this study were also categorised into three corresponding severity degrees.

The LAI-based relative threshold method mainly addresses four key issues as given below:

1. *Determination of monitoring time-windows for different crops*: To effectively assess agricultural drought impacts, all LAI time-windows of the major crops were determined to investigate the key phenological periods. To identify the phonologically most sensitive period for agricultural drought impacts, we constructed a series of candidate LAI time-windows using consecutive MODIS 8-day composites within the predefined growing season of each crop. These candidate windows do not represent distinct agronomic phenological stages, but rather computationally defined temporal windows used for optimization. For example, for autumn-harvest crops in the NEC-IMR, 153 candidate LAI time windows were generated from DOY 145 to DOY 273. Similar sets of candidate windows were constructed for other crops and regions based on their respective growing seasons (Tables S2–S7). Following threshold optimization, only one LAI time window that maximizes consistency with historical drought statistics is selected and referred to as the key phenological period. This selected window corresponds to a biologically meaningful growth stage (e.g., heading or grain filling) rather than representing multiple phenological stages.
2. *Selection of baseline year*: The LAI state of the baseline year was then used to quantify the relative degree of agricultural drought within the 2006–2020 study period. To ensure the baseline year as close as possible to no drought conditions, we selected the year during which agricultural drought impacts across all three severity degrees (drought-covered, drought-damaged, and crop failure) were the weakest. Based on the statistics of agricultural drought areas in BFDDC (Fig. S1), the baseline years were set as 2013 for Heilongjiang, Jilin, Liaoning and Inner Mongolia, 2020 for Shandong and Hebei, 2015 for Jiangsu, Henan and Anhui, 2008 for Sichuan, Chongqing and Hubei, and 2012 for Hunan and Jiangxi.
3. *Identification of the agricultural drought-impacted areas*: Using the drought-covered area in Hunan (consist of summer-harvest crops, autumn-harvest crops and early rice) as an example, the steps to identify agricultural drought-covered area by certain thresholds are explained as follows.
  First, for specific time-window of summer-harvest crops, the average LAI was calculated across this period in each year, producing yearly averaged LAI time series (*LAI*_*s*_). The average LAI values at the corresponding period in the baseline year, denoted as *LAI*_*sbase*_, served as the reference.
  Then, given certain thresholds *T*_*s*_ for identifying drought-covered area for summer-harvest crops. Extract the grids with their *LAI*_*s*_* ≤ *${(T}_{s}\,\times \,{{LAI}}_{{sb}{ase}})$ in each year as extracted drought-covered area for summer-harvest crops $\left. ({{EA}}_{s}\right)$. The drought-covered area for autumn-harvest crops (${{EA}}_{a}$) and early rice (${{EA}}_{e}$) are extracted by the same procedure of *LAI*_*a*_*≤* $({T}_{a}$ $\times \,{{LAI}}_{{ab}{ase}})$ and *LAI*_*e*_*≤* $({T}_{e}$ $\times \,{{LAI}}_{{eb}{ase}})$, respectively. The specific calculation equation are as flows:
  1 $${{EA}}_{s}={Rastersize}\times {\sum }_{i}^{n}{I({LAI}}_{s\_i}{\rm{\le }}({T}_{s}\times {{LAI}}_{{sb}{as}{e}_{\_i}}))$$
  2 $${{EA}}_{a}={Rastersize}\times {\sum }_{i}^{n}I({{LAI}}_{a\_i}{\rm{\le }}({T}_{a}\times {{LAI}}_{{ab}{ase}\_i}))$$
  3 $${{EA}}_{e}={Rastersize}\times {\sum }_{i}^{n}I({{LAI}}_{e\_i}{\rm{\le }}({T}_{e}\times {{LAI}}_{{eb}{ase}\_i}))$$
  where ${{LAI}}_{s{\_i}}$, ${{LAI}}_{a{\_i}}$ and ${{LAI}}_{e{\_i}}$ are the actual mean LAI value of summer-harvest crops, autumn-harvest crops and early rice in grid *i* at the specific period, respectively; ${{LAI}}_{{sb}{ase\_i}}$, ${{LAI}}_{{ab}{ase\_i}}$ and ${{LAI}}_{{eb}{ase\_i}}$ are the mean LAI value of summer-harvest crops, autumn-harvest crops and early rice in grid *i* at the specific period in the baseline year, respectively; *n* is the total number of grids in the study area, *I*() is the indicator function that returns 1 if the condition is true and 0 otherwise, Rastersize is 500 m × 500 m.
  Finally, $${{EA}}_{s}$$, $${{EA}}_{a}$$, $${{EA}}_{e}$$ and historical drought-covered area in the baseline year ($${{HA}}_{{base}})$$ were summed to generate the annual time series of estimated drought-covered area ($${EA})$$.
4. *Determination of the optimal thresholds and key phenological periods*: To achieve optimal thresholds and key phenological periods for different degrees of agricultural drought impacts, the thresholds *T*_*a*_, *T*_*s*_ and *T*_*e*_ were iteratively tested from 0 to 100%, with an increment of 1% in each time-window. For each time-window, the consistency between ${EA}$ and the historical drought-covered area *(HA)* was evaluated using the Pearson correlation coefficient (R) and root-mean-square error (RMSE). The optimal relative threshold was determined as the one yielding the highest R and lowest RMSE, indicating the best simulation accuracy. The time-windows corresponding to these optimal thresholds were designated as key phenological periods. R and RMSE were calculated using the following equations:

4 $$R=\frac{\sum \left(EA-\frac{\sum EA}{n}\right)\left(HA-\frac{\sum HA}{n}\right)}{\begin{array}{cc}\sqrt{\sum {\left(EA-\frac{\sum EA}{n}\right)}^{2}} & \sqrt{{\left(HA-\frac{\sum HA}{n}\right)}^{2}}\end{array}}$$

5 $${\rm{RMSE}}=\sqrt{\frac{1}{n}\sum {\left({EA}-{HA}\right)}^{2}}$$

where *n* represents the number of study years. A higher positive *R* value and a lower RMSE value indicate a better consistency between the *HA* and *EA*, signifying better predictive performance for the estimated drought-covered area.

**Fig. 3:**
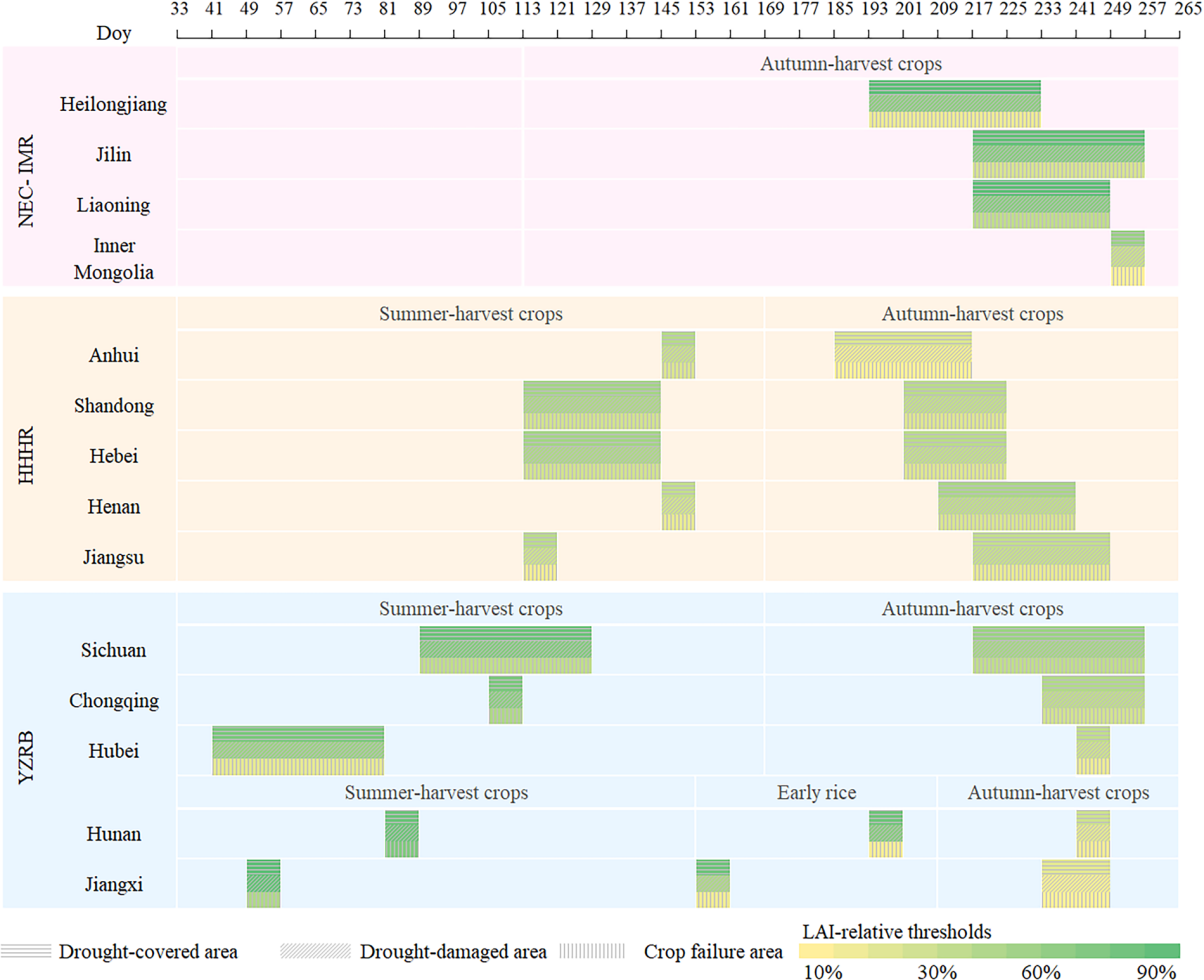
Optimal LAI-relative thresholds and the selected key phenological periods for each province in CMGR.

The thresholds for identifying drought-damaged area and crop failure area across the remaining provinces were determined using the same methodological framework described above. In the NEC-IMR, only the thresholds for autumn-harvest crops were determined due to their crop-specific phenological characteristics. For HHHR and Sichuan, Hubei and Chongqing within the YZRB region, thresholds for summer-harvest crops and autumn-harvest crops were determined. Following the above-mentioned approach, the optimal thresholds and key phenological periods are determined and presented in Fig. 3. Further details of the optimal thresholds of each province are provided in Table S8.

### Generation of the agricultural drought-impacted area dataset

By using the determined LAI-relative thresholds, the drought-covered area, drought-damaged area, and crop failure area for each province were extracted. Further, agricultural drought-impacted areas of all provinces were merged to generate the ADIA-CMGR. This comprehensive dataset includes the spatial extent of drought-covered, drought-damaged, and crop failure areas for autumn-harvest crops in NEC-IMR, autumn-harvest crops and summer-harvest crops in HHHR and some parts of YZRB (Sichuan, Chongqing and Hubei), and autumn-harvest crops, summer-harvest crops and early rice in the remaining parts of YZRB (Hunan and Jiangxi) over the period of 2006–2020.

**Fig. 4:**
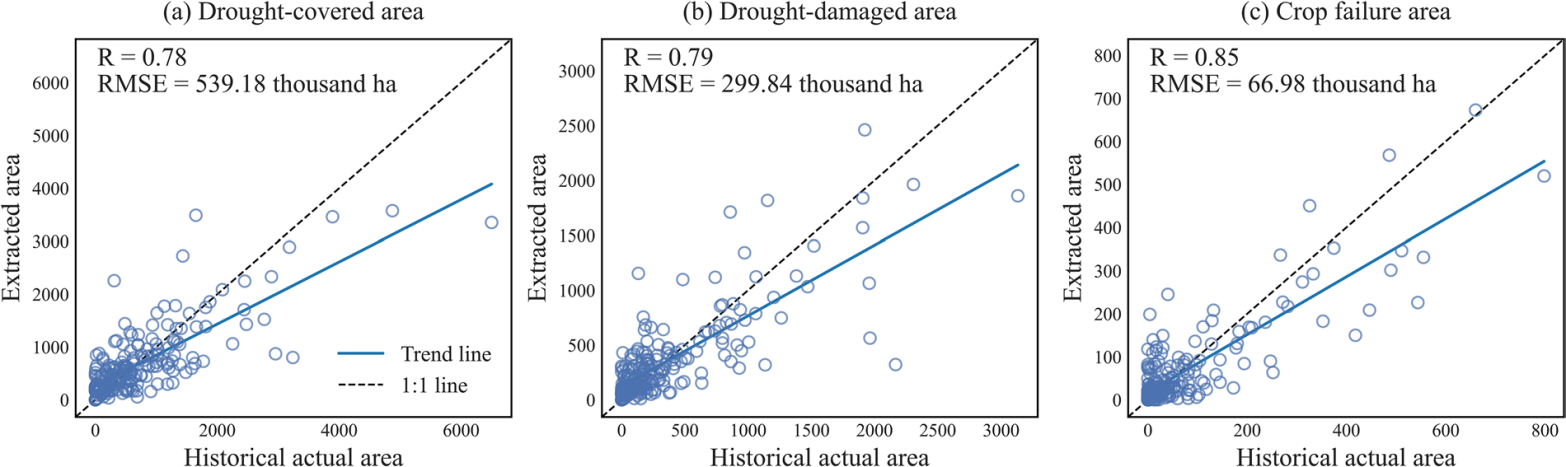
Scatter plots of the extracted and historical area for (**a**) drought-covered area, (**b**) drought-damaged area, and (**c**) crop failure area in the China’s main grain region.

**Fig. 5:**
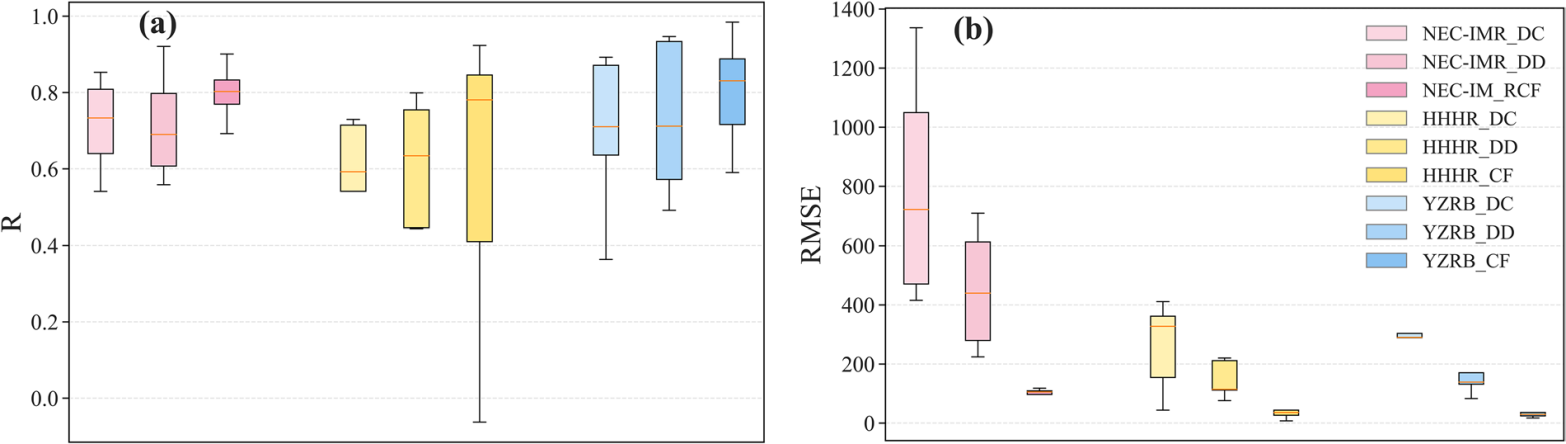
Box plots of (**a**) R and (**b**) RMSE for the extracted drought-covered area (DC), drought-damaged area (DD) and crop failure area (CF) in the Northeast China and Inner Mongolia region (NEC-IMR), the Huang-Huai-Hai region (HHHR) and the Yangtze River Basin (YZRB).

The comparison of drought-covered area, drought-damaged area, and crop failure area between extracted results and historical statistics for the study area and each sub-region are shown in Figs. 4, 5. The detailed comparisons of extracted results and historical statistics from BFFDC in each province are shown in Fig. S2. Overall, the results highlighted a good consistency between extracted and historical values, with R ranging between 0.78 to 0.85 and RMSE ranging between 66.98 to 539.18 thousand ha (Fig. 4). The crop failure area exhibits the highest accuracy, followed by drought-damaged area and drought-covered area. The progressive accuracy may be due to the pronounced LAI anomalies observed under severe drought conditions, resulting more precise identification of agricultural drought-impacted areas.

For NEC-IMR, the extracted agricultural drought-impacted areas exhibit strong agreement with historical statistics, with R ranging from 0.54 to 0.92 across the three drought degrees. It indicates that the extracted agricultural drought-impacted areas effectively capture the temporal dynamics of actual agricultural drought impacts (Fig. 5). Conversely, RMSE values in NEC-IMR (Ranging from 118.04 to 1336.58 thousand ha) are relatively higher compared to other regions. This is primarily due to the prevalence of agricultural droughts in NEC-IMR, where the annual average drought-covered area exceeds 1200 thousand ha.

For HHHR, the consistency of extracted agricultural drought-impacted areas is relatively lower, as envisaged from the R range of −0.06 to 0.84 across the three drought severity degrees. This reduced consistency could may stem from discrepancies between the historical data and extracted results in the Shandong and Anhui. In Shandong, the inconsistency is primarily due to the bias in the historical statistics for actual crop failure area in 2015, which cannot fully capture the actual agricultural drought condition in this region, as documented by other research^26^. Meanwhile, spanning over two different climatic districts in China (northern China and southern China), the complex agroclimatic conditions in Anhui pose a significant challenge for agricultural drought monitoring. Wu^37^ showed that the yield of winter wheat in Anhui exhibited weak correlations with drought indices, suggesting poor sensitivity of yield to drought in this region; hence, the extracted agricultural drought-impacted areas perform poorly (Fig. S2).

For YZRB, despite the overall agreement between extracted and historical areas is evident in Fig. 5, the accuracy of extracted results exhibits marked spatial variation across the region. The approach performed exceptionally well in extracting agricultural drought-impacted areas in Sichuan, Chongqing, and Hubei, with R ranging from 0.71 to 0.98 and RMSE ranging from 7.29 to 303.44 thousand ha. However, the accuracy is relatively lower in Hunan and Jiangxi, as depicted from R and RMSE estimates in the range of 0.36 to 0.71 and 29.44 to 387.92 thousand ha, respectively. This reduced performance is primarily due to the highly diverse crop planting structure and complex phenological patterns in these two provinces. According to the provincial statistical yearbook^38,39^, nearly ten crop types, including early-season rice, middle-season rice, late-season rice, rapeseeds, corn, and other oil-bearing crops, are cultivated across the growing season spanning the entire year. Meanwhile, the spatial distribution of these crops is highly heterogeneous and discrete, making it challenging to identify key phenological periods and thresholds across the province without detailed crop-specific agricultural land use maps. Moreover, the intense temporal variation of historical agricultural drought-impacted areas amplified the difficulty in the extraction of agricultural drought-impacted areas in these two provinces.

## Data Records

The complete ADIA-CMGR dataset is stored in the ZENODO repository and is available at https://zenodo.org/records/17940187^40^. The dataset is provided at 500 m resolution over the period of 2006–2020. The dataset contains a main folder called as *“01_TARGET3”* and an accompanying metadata file called as *“metadata.docx”*. The main folder stores 180 TIFF files accompanied by standard auxiliary files (.tfw,.aux.xml,.ovr,.vat.dbf,.xml) for georeferencing and attribute information. There are nine TIFF files corresponding to the drought-covered area, drought-damaged area, and crop failure area for autumn-harvest crops, summer-harvest crops, and early rice for each year. In the TIFF files, pixel values are classified as follows: 1: drought-covered area, 2: drought-damaged area, 3: crop failure area, and 99: invalid data.

Each tiff file provides the information of a specific drought degree for one crop type each year. Tiff file names follow the format “{Crop Type}_{Drought Degree}_{Year}.tif”, where Crop Type includes different sub-classes like Autumn (autumn-harvest crops), Summer (summer-harvest crops), and ER (early rice); Drought Degree denotes the drought impact degree (DC: drought-covered, DD: drought-damaged, CF: crop failure); and Year indicates the corresponding year for the period 2006–2020.

## Technical Validation

### Rationality of the determined optimal thresholds and key phenological periods

Overall, the determined value of optimal thresholds (see Fig. 3) decreases from drought-covered area to drought-damaged area and further to crop failure area, which is in line with the progressive severity of the three degrees of agricultural drought impacts^41,42^. Spatially, the thresholds for each crop at a given drought degree are relatively consistent between the NEC-IMR and HHHR regions, but higher variability is observed in the YZRB, resulting from the complex cropping systems across different provinces within this region. Furthermore, LAI thresholds in NEC-IMR are relatively larger compared to other regions, indicating that LAI anomalies are relatively smaller in NEC-IMR than in other regions under the same degree of agricultural drought impacts. This observation is consistent with findings from previous studies, which indicate that crops in NEC-IMR exhibit greater sensitivity to drought stress, largely because drought is a predominant limiting factor for crop biomass in this area^43,44^.

The key phenological periods identified in this study (Fig. 3) are mostly in line with the heading and milk stages of rice, wheat, maize, and the podding stage of soybean^33,45,46^. Overall, these stages are the critical phenological periods for the formation of grain spike and grain weight of the crops, in which nutrients are quickly transported to the seed, accumulated, and the dry matter reaches its peak. The lack of water supply in these stages would significantly affect the growth of kernels and further cause the reduction of crop yield^43,44^. Moreover, the LAI of healthy crops are relatively high in these stages, which are more easily differentiated from the LAI of drought-affected crops in harvest stages. After the milk stage, the plants begin to senesce, and the influence of water stress on yield becomes progressively less significant^47^. Spatially, the key phenological periods of summer-harvest crops in YZRB occur earlier than those in the HHHR, primarily due to the different growing seasons of main crops in these two regions. Winter rapeseeds are one of the main summer-harvest crops in YZRB, with its heading and mature stage occurring half a month earlier than winter wheat, the dominant summer-harvest crop in HHHR^48,49^. Similarly, the key phenological periods of autumn-harvest crops in HHHR are earlier than the key phenological periods in YZRB, pointing to the earlier heading and mature stage of summer maize in HHHR than late rice in YZRB^33^. Hence, the spatial and temporal adaptability of these determined LAI-relative thresholds and key phenological periods among the three sub-regions of CMGR supports the validity and reliability of the generated dataset.

### Spatial validation by text-based agricultural drought reports

**Fig. 6:**
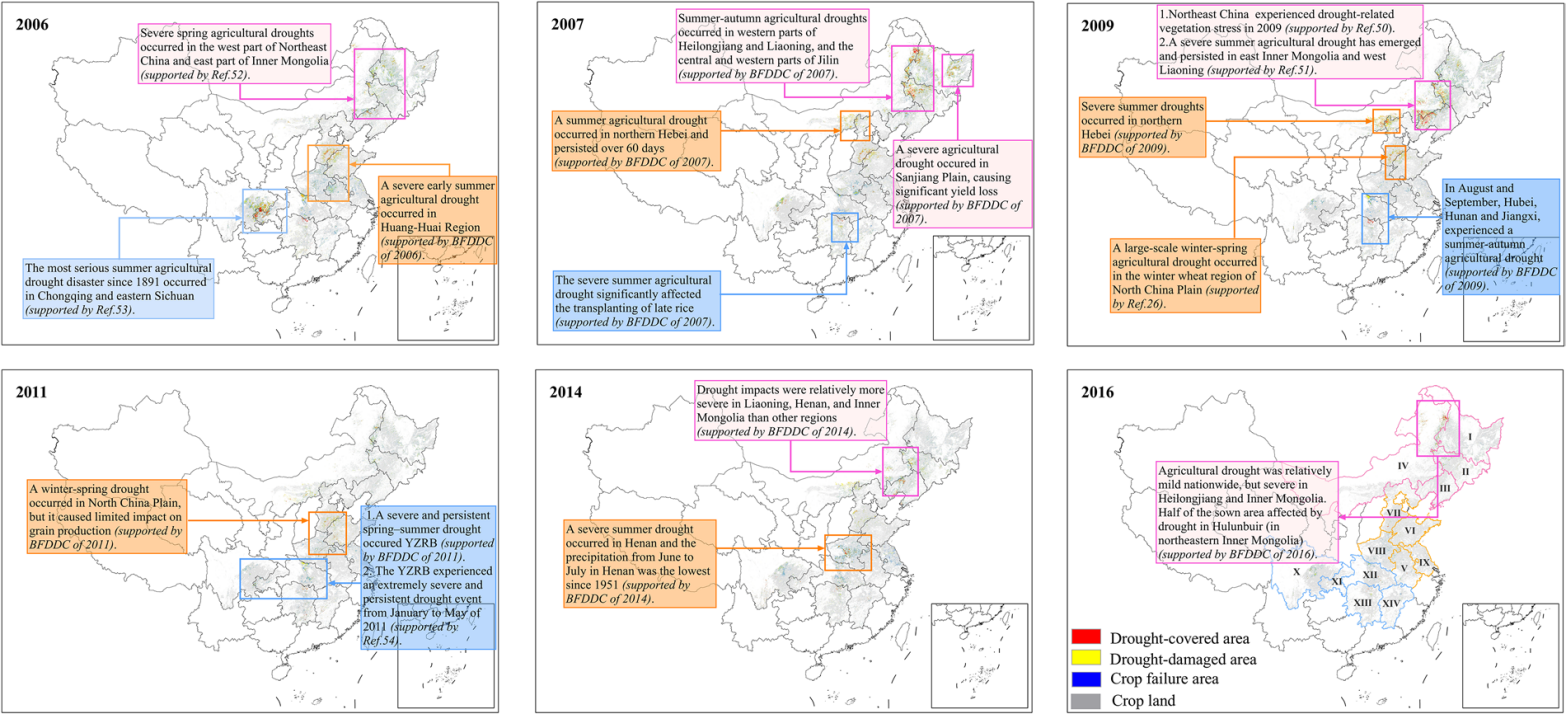
Distribution of extracted agricultural drought-impacted areas and the text-based supporting evidence in typical drought years. The specific names of each province in CMGR are provided in Fig. 1.

The extracted drought-covered area, drought-damaged area, and crop failure area of summer-harvest crops, autumn-harvest crops and early rice in the CMGR during 2006–2020 are shown in Figs. S3–S5. Due to the limited availability of quantitative drought impact data, we further validate the accuracy of ADIA-CMGR through the text-based drought descriptions of agricultural drought impacts sourced from the BFFDC and other existing literature. The spatial distribution of the extracted agricultural drought-impacted areas during the six most severe drought years (2006, 2007, 2011, 2014 and 2016) were validated on a year-by-year basis. Supporting evidence and documentation for agricultural drought-impacted areas in CMGR are provided in Fig. 6. Detailed analysis about the rationality of extracted agricultural drought-impacted areas in each sub-region is discussed as follows.

NEC-IMR suffered the most widespread agricultural droughts in 2007, 2009 and 2016, resulting in autumn-harvest crops being damaged severely. The extracted agricultural drought-impacted areas are mainly concentrated in the western part of Heilongjiang, Jilin, and Liaoning, as well as the southern and eastern parts of Inner Mongolia (Fig. S3). These findings are consistent with the description of agricultural drought impacts in BFFDC, which reported a severe and persistent summer-to-autumn agricultural drought in 2007 across western Heilongjiang, central and western Jilin, and western Liaoning. Besides, severe agricultural droughts have also been demonstrated to have emerged and persisted in eastern Inner Mongolia, western Heilongjiang, and western Liaoning in 2009 and 2016 by a previous study, resulting in a substantial area affected by drought in NEC-IMR^50,51^.

HHHR experienced severe agricultural droughts in 2006, 2009 and 2014 (Figs. S3,S4). The agricultural drought-impacted areas in this region are mainly concentrated in the northwestern and southeastern parts of Hebei, the northwestern part of Shandong, and the eastern part of Henan. As illustrated in Fig. 6, similar findings have been reported by a previous study that severe summer agricultural droughts were widespread in the Huang-Huai region in 2006, driven by insufficient precipitation, rising temperature, and frequent windy weather, adversely affecting the summer-harvest crops^52^. Furthermore, according to the BFDDC, Hebei was significantly affected by drought due to cumulative winter precipitation in central and southern parts reaching only 20% of the average annual rainfall in 2008. Similarly, Henan experienced a severe summer drought in 2014, with precipitation from June to July falling to its lowest level since 1951, resulting in substantial drought-impacted areas in the corresponding year. These documented events align well with the prevalent agricultural drought-impacted areas extracted in southern Hebei in 2009 and central Henan in 2014.

Severe droughts were observed across the YZRB in 2006, 2007, and 2011, with the drought-impacted areas mainly concentrated in the eastern part of Sichuan, the western part of Chongqing, and the central part of Hubei. It is widely acknowledged that the Sichuan-Chongqing region suffered the most severe drought scenario in recent 50 years during 2006–2007, causing substantial losses in crop yield^53^. Similarly, extensive drought impacted area were also detected in Sichuan and Chongqing during 2006 to 2007 in this study. Furthermore, the rationality of the extensive drought-impacted areas as extracted in 2011 are also evidenced by the widespread and persistent spring–summer drought across YZRB in 2011 as adopted from another study^54^. Overall, the extracted agricultural drought-impacted areas show high consistency with the text-based records of agricultural drought impacts in each sub-region, demonstrating the acceptable accuracy, precise temporal variability and reasonable spatial distribution of the generated dataset.

### Limitations

This novel dataset elucidates the spatial pattern of agricultural drought-impacted areas across different harvest seasons, which can serve as a robust tool for formulating effective drought response strategies and guiding fine-scale agricultural practices. Despite the comprehensive analysis approach, the ADIA-CMGR dataset is subject to a few limitations:

1. LAI was chosen as the primary agricultural drought index to identify agricultural drought-impacted areas in this study. This choice reflects the study’s focus on capturing the ultimate consequences of agricultural droughts, particularly in terms of the total area experiencing varying degrees of yield reduction. While numerous drought indices integrating hydrological, meteorological, and vegetation attributes are commonplace in practical applications, vegetation drought indices were considered as the most direct driver to monitor actual crop growth conditions. Among those vegetation indices, LAI stands out as a robust vegetation index and are more sensitive to the variation in crop yield than others. Although LAI is strongly related to the drought-caused yield loss, its values can also be influenced by other stressors such as pest infestations and natural disasters. These confounding factors may obscure the true consequences of drought impacts, potentially affecting the accuracy of agricultural drought detection. Meanwhile, due to the limited availability of LAI data sources, the ADIA-CMGR dataset was generated at 500 m resolution. At this moderate resolution, mixed pixels of LAI may blur crop-specific drought signals and introduce uncertainties in identifying and quantifying drought impact area. Future research will aim to disentangle these influences through more quantitative methods and incorporate multiple high-resolution drought indices. This multivariate approach is expected to enhance the precision and spatial resolution of agricultural drought impact assessments.
2. Uncertainties also revolve around the settings of the adopted methodology. Firstly, the phenological periods and thresholds across diverse crop types were set as the uniformed application. Due to the absence of detailed crop-specific coverage data, crops in this region were classified into summer-harvest crops, autumn-harvest crops, and early rice for extracting their respective drought-impacted areas. Consequently, the thresholds and key phenological periods are determined for these three crop types. Despite the growing seasons being generally constant, differences still exist among the phenological periods of different autumn-harvest crops (summer-harvest crops), such as late rice and spring maize. The potential influence on the generated ADIA-CMGR dataset is expected to be limited given the predominance of simple cropping systems and a consistent phenological period but may arouse across diverse crop types in YZRB. Secondly, the baseline was set as the drought state of a specific year during which agricultural drought impacts across all three severity degrees are weakest. The rationale behind this choice is rooted in ensuring the baseline closest to no-drought conditions, thereby minimizing potential bias in the relative assessment of drought severity, arising from the inherent agricultural drought degree of baseline. However, despite having the smallest drought-impacted area, the baseline year does not represent a truly drought-free condition, inevitably causing certain overestimation of threshold. We would like to continuously incorporate more detailed crop coverage data and observed crop physiological data to reduce uncertainty in the drought impact assessment.
3. Given the limited publicly available data on agricultural drought impacts, this study relies on the text-based agricultural drought impact information acquired from BFDDC and published literatures to generate and validate ADIA-CMGR. The statistical agricultural drought-impacted areas in BFDDC effectively capture the temporal fluctuations of agricultural drought impacts well. However, inaccuracies may inevitably arise and accumulate as the data is transferred into a broader spatiotemporal context. Such inconsistencies may propagate through the spatiotemporal integration process and potentially lead to deviations in the identification and mapping of agricultural drought-impacted areas in specific years^18^. Despite modest uncertainties existing in statistical agricultural drought-impacted areas, the substantial spatial coherence observed between the extracted agricultural drought-impacted areas and text-based records over the entire analysis period substantiates the reliability of the generated dataset.

## Usage Notes

Agricultural drought impact data enable the transition from hazard-based to impact-based drought information, providing not only the likelihood of drought occurrence, but also anticipating the severity and spatial extent of its expected impacts on crops in the coming months^9,55^. The notion of anthropogenic drought^56^ underscores that droughts are not solely driven by natural climate variability, but can also be caused or intensified by human activities, such as the expansion of agriculture beyond the carrying capacity of local water resources. In this context, high-resolution agricultural drought impact data represent a major advancement, as they allow for the integration of climate extremes with human-induced dimensions of drought.

The ADIA-MGPRC dataset generated in this study provides 500 m resolution maps of agricultural drought-impacted areas from 2006 to 2020, including drought-covered area, drought-damaged area, and crop failure area for summer-harvest crops, autumn-harvest crops, and early rice. The dataset is designed to support regional- to basin-scale analyses of agricultural drought impacts and their spatial–temporal variability. Potential applications include agricultural drought risk assessment, analysis of drought impact recurrence, evaluation of cropping system vulnerability, model calibration and validation for drought monitoring, and support for drought mitigation and agricultural planning.

Users should note that drought severity classes are derived from LAI-based relative thresholds and represent inferred drought impacts rather than direct yield loss measurements. Crop types are generalized into three major categories; therefore, applications in regions with highly diverse or rapidly changing cropping systems should be interpreted with caution. As LAI can also respond to non-drought stressors, users are encouraged to integrate complementary information such as meteorological drought indices, soil moisture, or yield statistics when attributing impacts specifically to drought. Future updates may further refine the dataset as more detailed crop and phenological information becomes available.

## Supplementary information

Supplment

## Data Availability

The complete ADIA-CMGR dataset is stored in the ZENODO repository and is available at https://zenodo.org/records/17940187 under the Creative Commons Attribution 4.0 International license.

## Code availability

The MATLAB code used to generate the agricultural drought-impacted area dataset in the China’s main grain region is available to the public at https://github.com/SJL-UCAS/ADAD_generate.
