# Supplementary material for: A 500-m Agricultural Drought Impact Dataset in China’s Main Grain Region: Toward Impact-Based Drought Monitoring
Source: Sci Data. 2026 Feb 5;13:357. doi: 10.1038/s41597-026-06732-3 (PMC12982602; doi:10.1038/s41597-026-06732-3)
Supplement: Supplementary file 1 — Supplment [file 41597_2026_6732_MOESM1_ESM.docx]

**A 500-m Agricultural Drought Impact Dataset in China’s Main Grain Region: Toward Impact-Based Drought Monitoring**

Jiali Shi^1,2^, Yan-Fang Sang^1,2,3^, Amir AghaKouchak^4,5^, Sonam Sandeep Dash^6^, Faith Ka Shun Chan^7^,

^1^Key Laboratory of Water Cycle & Related Land Surface Processes, Institute of Geographic Sciences and Natural Resources Research, Chinese Academy of Sciences, Beijing 100101, China

^2^University of Chinese Academy of Sciences, Beijing 101407, China

^3^Key Laboratory of Compound and Chained Natural Hazards, Ministry of Emergency Management of China, Beijing 100085, China

^4^Department of Civil and Environmental Engineering, University of California Irvine, CA 92697, USA

^5^United Nations University Institute for Water, Environment and Health (UNU-INWEH), Ontario, Canada.

^6^Physical Science and Engineering Division, King Abdullah University of Science and Technology, Saudi Arabia

^7^School of Geographical Sciences, Faculty of Science and Engineering, University of Nottingham Ningbo China, Ningbo 315100, China

*Correspondence to:* Yan-Fang Sang (sangyf@igsnrr.ac.cn; [sunsangyf@gmail.com](mailto:sunsangyf@gmail.com))

**This file contents:**

- Figure S1 Temporal variations of agricultural drought-impacted areas of the Northeast China and Inner Mongolia region (NEC-IMR), the Huang-Huai-Hai region (HHHR) and the Yangtze River Basin (YZRB).
- Figure S2 Comparison of the historical and extracted agricultural drought-impacted areas in each province of the NEC-IMR, the HHHR and the YZRB.
- Figure S3 Spatial distribution of the extracted agricultural drought areas for autumn-harvest crops in the China’s main grain region during 2006-2020.
- Figure S4 Spatial distribution of the extracted agricultural drought areas for summer-harvest crops in the China’s main grain region during 2006-2020.
- Figure S5 Spatial distribution of the extracted agricultural drought areas for early rice in Hunan and Jiangxi during 2006-2020.
- Tables S1 Main crops and their phenological calendars in the NEC-IMR, the HHHR and the YZRB.
- Tables S2 Start and end time of 153 time-windows for autumn-harvest crops in the NEC-IMR
- Tables S3 Start and end time of 105 time-windows for summer-harvest crops in the HHHR
- Tables S4 Start and end time of 75 time-windows for autumn-harvest crops in the HHHR
- Tables S5 Start and end time of 78 time-windows for summer-harvest crops in the YZRB
- Tables S6 Start and end time of 78 time-windows for autumn-harvest crops in the YZRB
- Tables S7 Start and end time of 21 time-windows for early rice in the YZRB
- Tables S8 The optimal thresholds of each province in the NEC-IMR, the HHHR and the YZRB

**
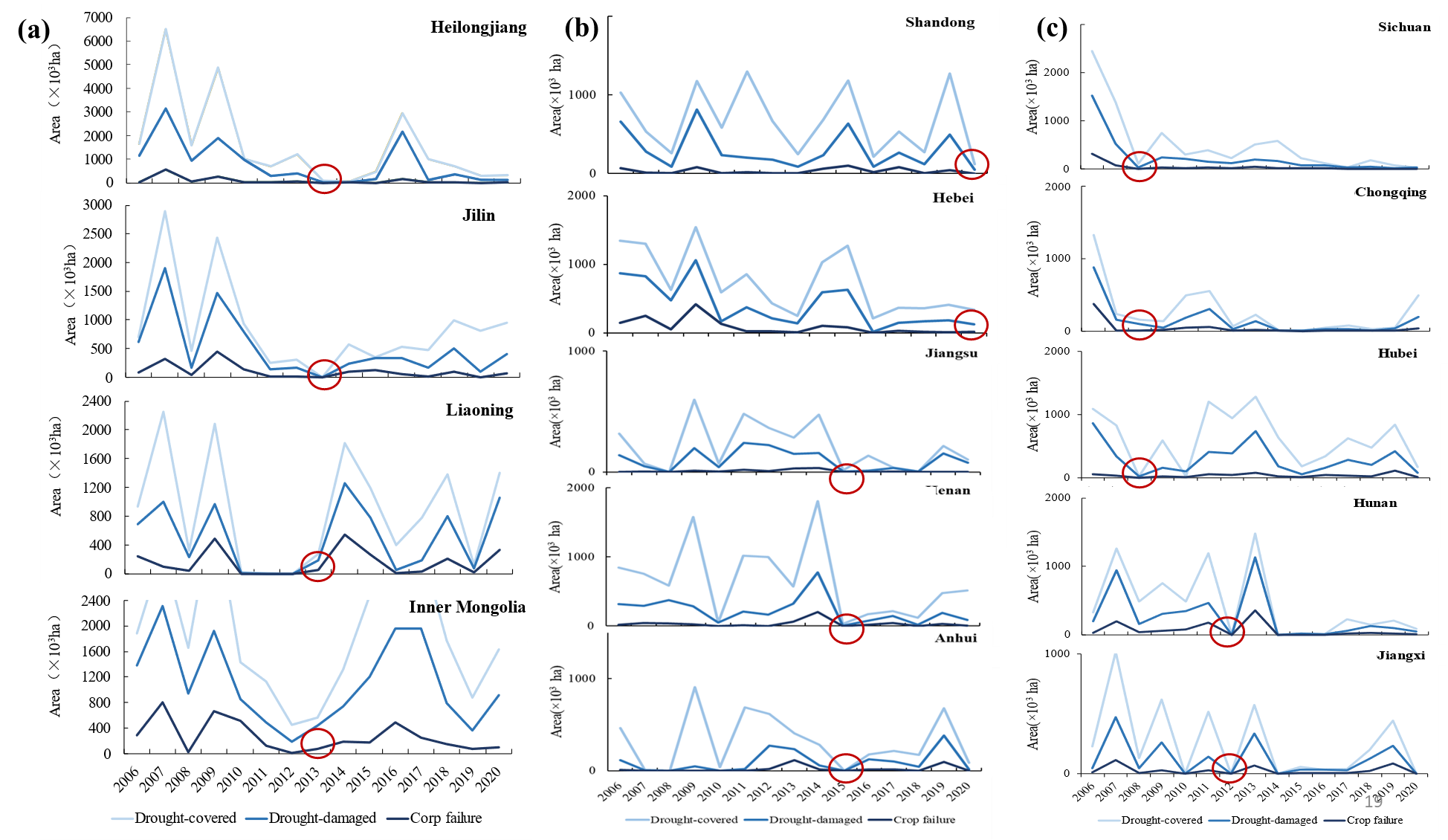
**

Fig. S1 Temporal variations of agricultural drought-impacted areas of the Northeast China and Inner Mongolia region (NEC-IMR), the Huang-Huai-Hai region (HHHR) and the Yangtze River Basin (YZRB).


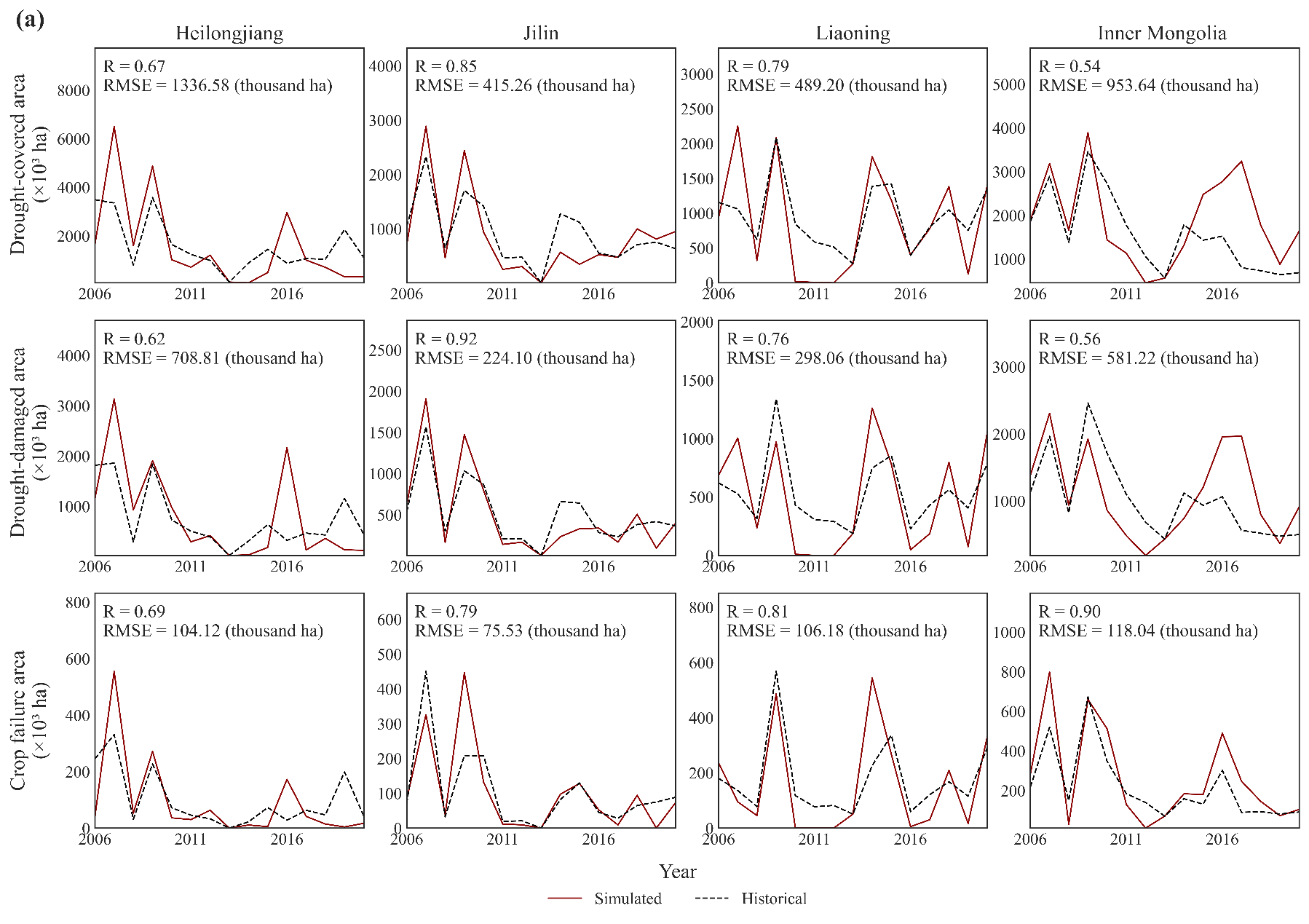

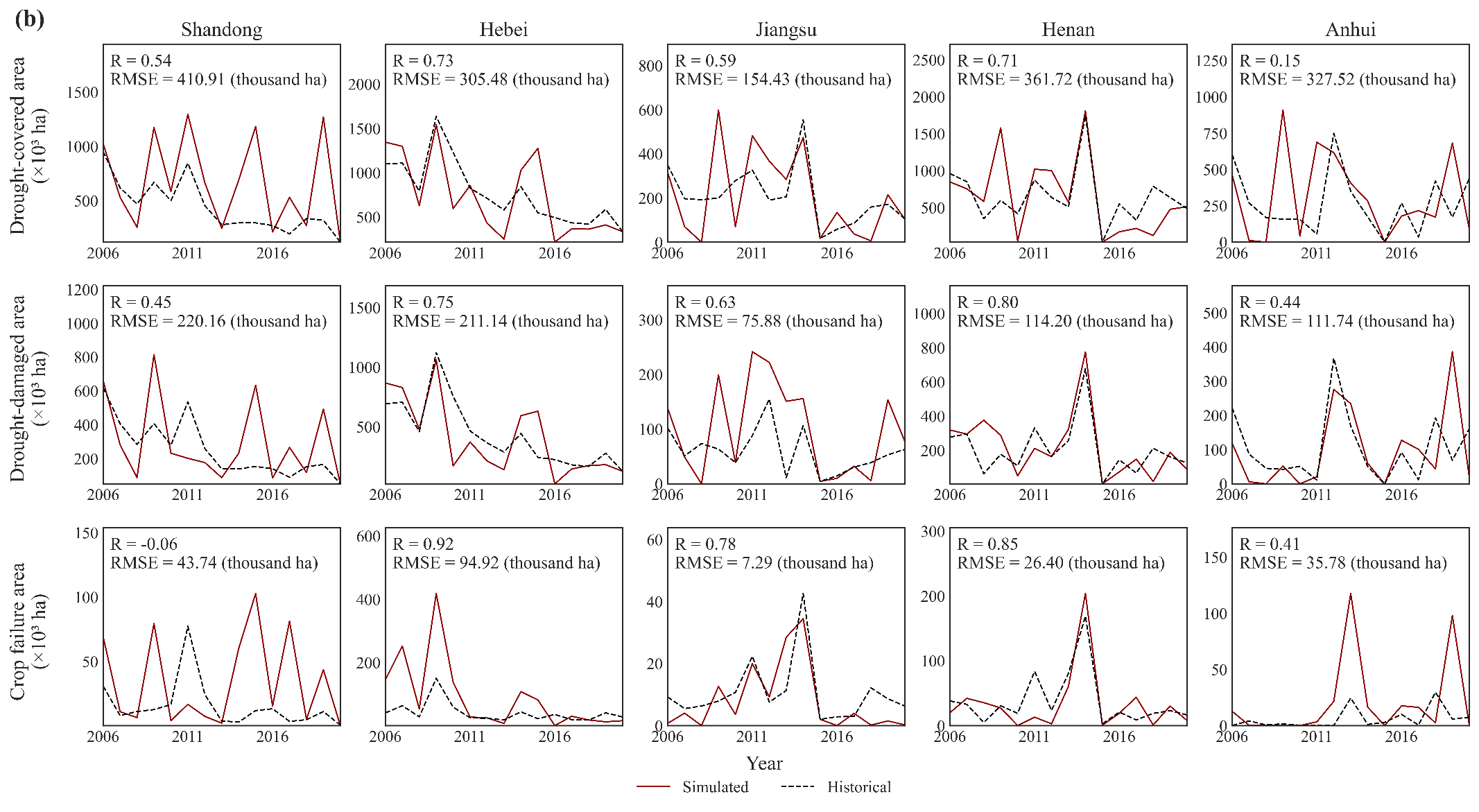

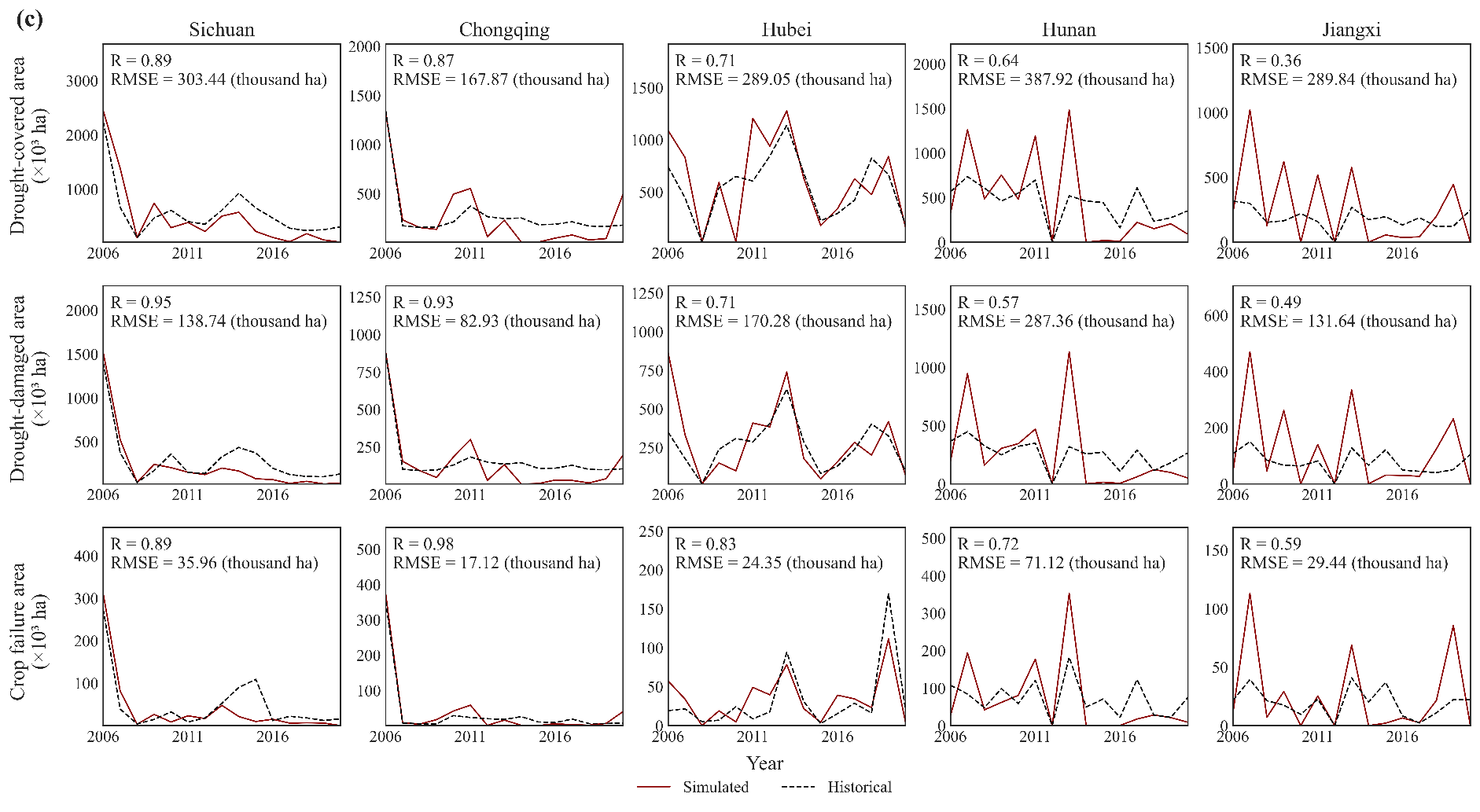


Fig. S2 Comparison of the historical and simulated agricultural drought-impacted areas in each province of the NEC-IMR, the HHHR and the YZRB.


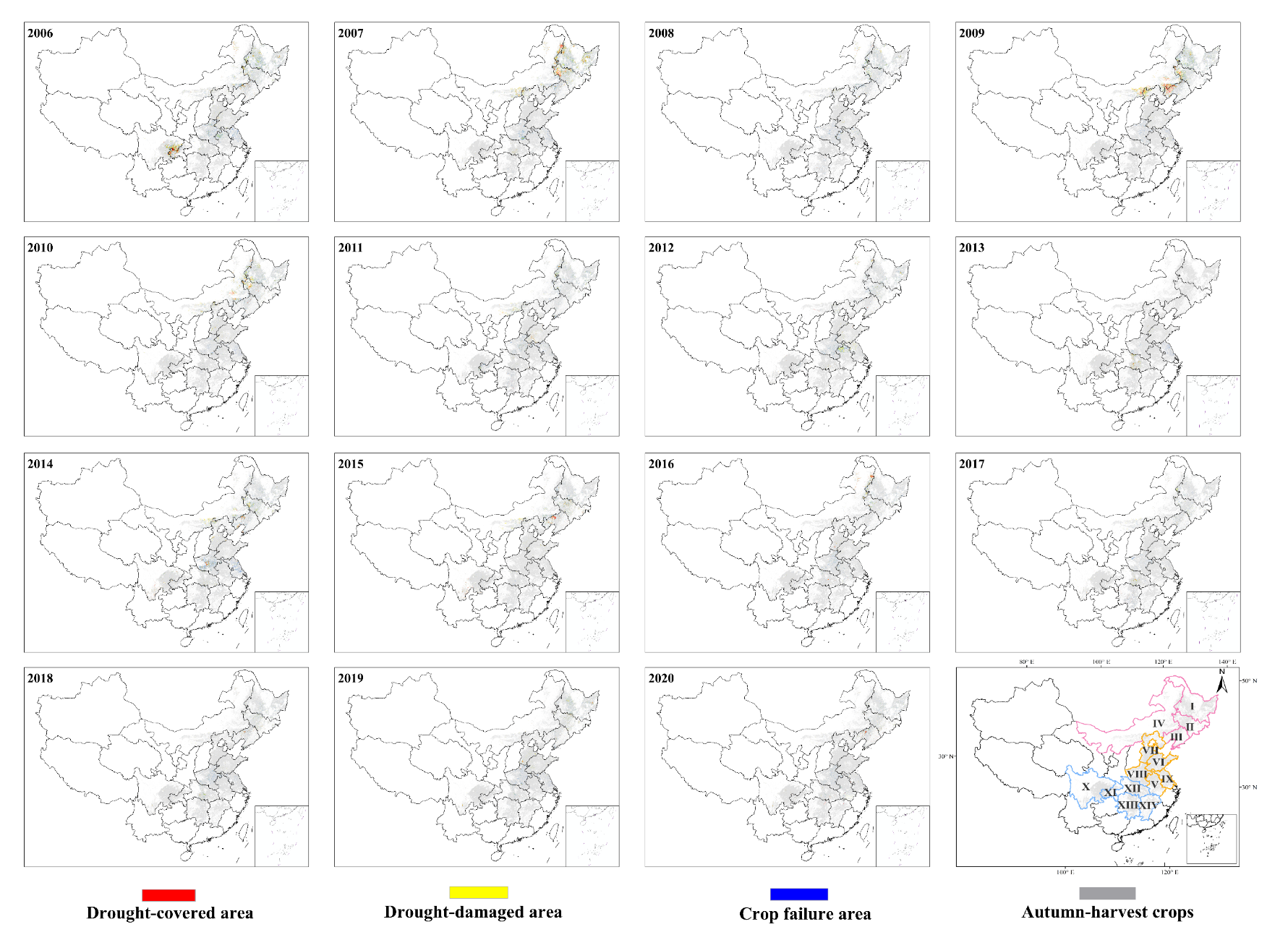


- Fig. S3. Spatial distribution of the extracted agricultural drought areas for autumn-harvest crops in the China’s main grain region during 2006-2020.

during 2006-2020.


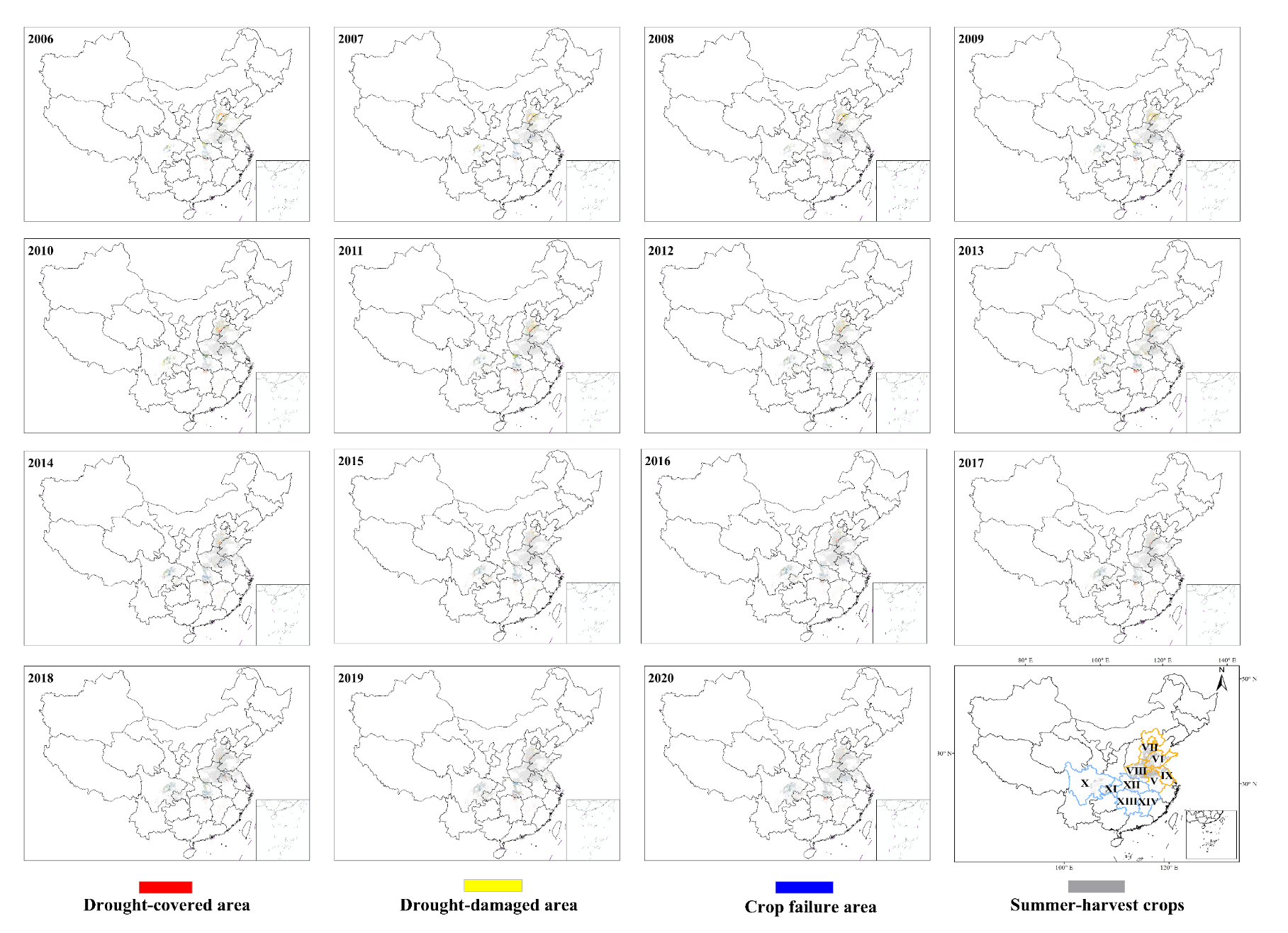


- Fig. S4. Spatial distribution of the extracted agricultural drought areas for summer-harvest crops in the China’s main grain region during 2006-2020.

during 2006-2020.


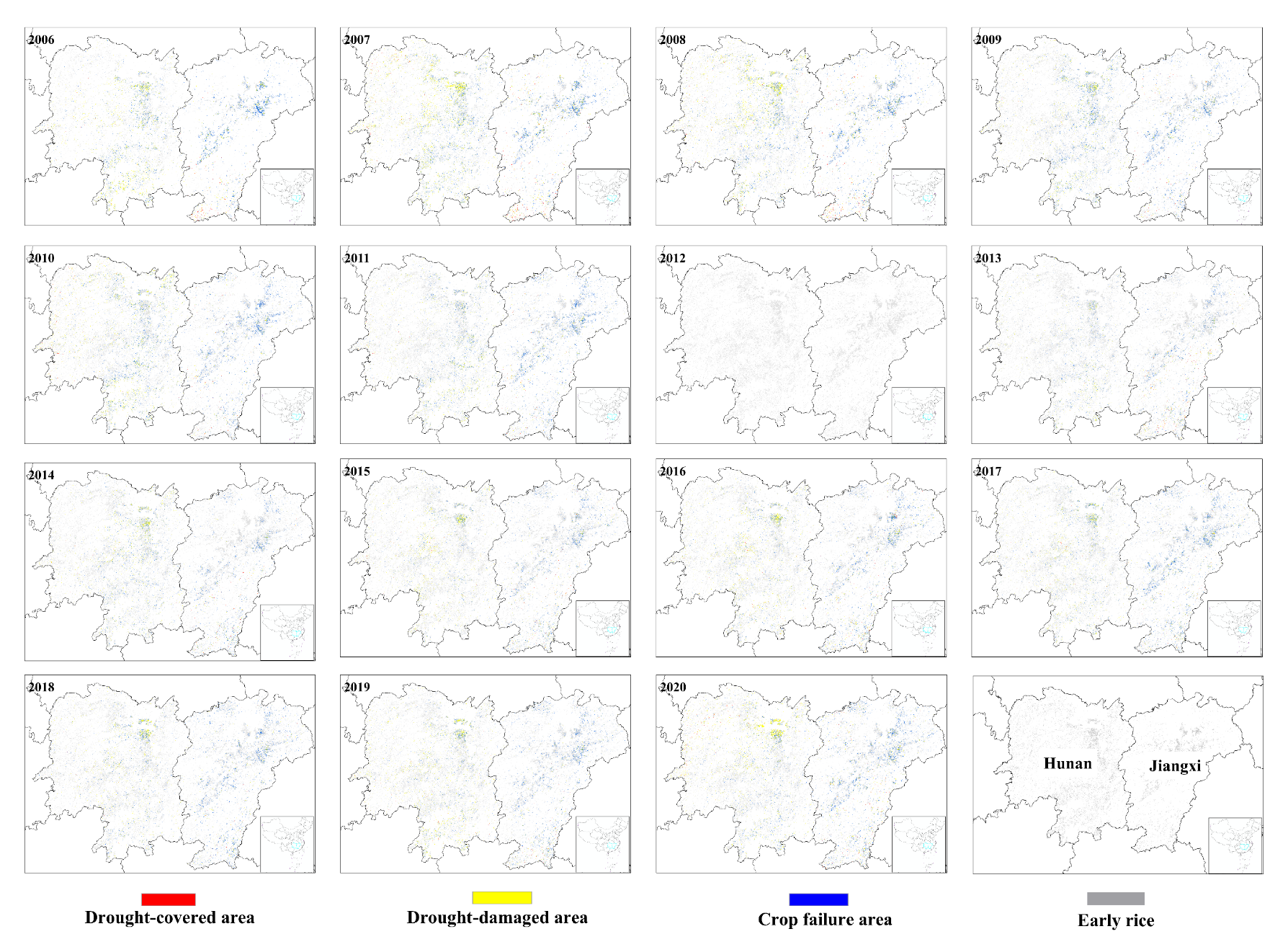


Fig. S5. Spatial distribution of the extracted agricultural drought areas for early rice in Hunan and Jiangxi during 2006-2020.

Tab. S1 Main crops and their phenological calendars in the NEC-IMR, the HHHR and the YZRB. The green cells represent the summer-harvest crops, the yellow cells represent the autumn-harvest crops, and the orange cells represent the early rice.

|  | Month | 9 | | | 10 | | | 11 | | | 12 | | | 1 | | | 2 | | | 3 | | | 4 | | | 5 | | | 6 | | | 7 | | | 8 | | | 9 | | | 10 | | |
| --- | --- | --- | --- | --- | --- | --- | --- | --- | --- | --- | --- | --- | --- | --- | --- | --- | --- | --- | --- | --- | --- | --- | --- | --- | --- | --- | --- | --- | --- | --- | --- | --- | --- | --- | --- | --- | --- | --- | --- | --- | --- | --- | --- |
|  | Ten-day | 1 | 2 | 3 | 1 | 2 | 3 | 1 | 2 | 3 | 1 | 2 | 3 | 1 | 2 | 3 | 1 | 2 | 3 | 1 | 2 | 3 | 1 | 2 | 3 | 1 | 2 | 3 | 1 | 2 | 3 | 1 | 2 | 3 | 1 | 2 | 3 | 1 | 2 | 3 | 1 | 2 | 3 |
| NEC-IM | Single rice |  |  |  |  |  |  |  |  |  |  |  |  |  |  |  |  |  |  |  |  |  |  |  |  |  |  |  |  |  |  |  |  |  |  |  |  |  |  |  |  |  |  |
|  | Spring maize |  |  |  |  |  |  |  |  |  |  |  |  |  |  |  |  |  |  |  |  |  |  |  |  |  |  |  |  |  |  |  |  |  |  |  |  |  |  |  |  |  |  |
|  | Soybean |  |  |  |  |  |  |  |  |  |  |  |  |  |  |  |  |  |  |  |  |  |  |  |  |  |  |  |  |  |  |  |  |  |  |  |  |  |  |  |  |  |  |
| HHH | Winter wheat |  |  |  |  |  |  |  |  |  |  |  |  |  |  |  |  |  |  |  |  |  |  |  |  |  |  |  |  |  |  |  |  |  |  |  |  |  |  |  |  |  |  |
|  | Spring wheat |  |  |  |  |  |  |  |  |  |  |  |  |  |  |  |  |  |  |  |  |  |  |  |  |  |  |  |  |  |  |  |  |  |  |  |  |  |  |  |  |  |  |
|  | Summer maize |  |  |  |  |  |  |  |  |  |  |  |  |  |  |  |  |  |  |  |  |  |  |  |  |  |  |  |  |  |  |  |  |  |  |  |  |  |  |  |  |  |  |
|  | Spring maize |  |  |  |  |  |  |  |  |  |  |  |  |  |  |  |  |  |  |  |  |  |  |  |  |  |  |  |  |  |  |  |  |  |  |  |  |  |  |  |  |  |  |
|  | Single rice |  |  |  |  |  |  |  |  |  |  |  |  |  |  |  |  |  |  |  |  |  |  |  |  |  |  |  |  |  |  |  |  |  |  |  |  |  |  |  |  |  |  |
| YZRB | Early rice |  |  |  |  |  |  |  |  |  |  |  |  |  |  |  |  |  |  |  |  |  |  |  |  |  |  |  |  |  |  |  |  |  |  |  |  |  |  |  |  |  |  |
|  | Single rice |  |  |  |  |  |  |  |  |  |  |  |  |  |  |  |  |  |  |  |  |  |  |  |  |  |  |  |  |  |  |  |  |  |  |  |  |  |  |  |  |  |  |
|  | Late rice |  |  |  |  |  |  |  |  |  |  |  |  |  |  |  |  |  |  |  |  |  |  |  |  |  |  |  |  |  |  |  |  |  |  |  |  |  |  |  |  |  |  |
|  | Spring maize |  |  |  |  |  |  |  |  |  |  |  |  |  |  |  |  |  |  |  |  |  |  |  |  |  |  |  |  |  |  |  |  |  |  |  |  |  |  |  |  |  |  |
|  | Winter wheat |  |  |  |  |  |  |  |  |  |  |  |  |  |  |  |  |  |  |  |  |  |  |  |  |  |  |  |  |  |  |  |  |  |  |  |  |  |  |  |  |  |  |
|  | Rapeseed |  |  |  |  |  |  |  |  |  |  |  |  |  |  |  |  |  |  |  |  |  |  |  |  |  |  |  |  |  |  |  |  |  |  |  |  |  |  |  |  |  |  |

Tab. S2 Start and end time of 153 time windows for autumn-harvest crops in NEC-IMR

| End  (DOY) | Start (DOY) | | | | | | | | | | | | | | | | |  |
| --- | --- | --- | --- | --- | --- | --- | --- | --- | --- | --- | --- | --- | --- | --- | --- | --- | --- | --- |
|  | 145 | 153 | 161 | 169 | 177 | 185 | 193 | 201 | 209 | 217 | 225 | 233 | 241 | 249 | 257 | 269 | 273 | |
| 145 | 1 |  |  |  |  |  |  |  |  |  |  |  |  |  |  |  |  | |
| 153 | 2 | 3 |  |  |  |  |  |  |  |  |  |  |  |  |  |  |  | |
| 161 | 4 | 5 | 6 |  |  |  |  |  |  |  |  |  |  |  |  |  |  | |
| 169 | 7 | 8 | 9 | 10 |  |  |  |  |  |  |  |  |  |  |  |  |  | |
| 177 | 11 | 12 | 13 | 14 | 15 |  |  |  |  |  |  |  |  |  |  |  |  | |
| 185 | 16 | 17 | 18 | 19 | 20 | 21 |  |  |  |  |  |  |  |  |  |  |  | |
| 193 | 22 | 23 | 24 | 25 | 26 | 27 | 28 |  |  |  |  |  |  |  |  |  |  | |
| 201 | 29 | 30 | 31 | 32 | 33 | 34 | 35 | 36 |  |  |  |  |  |  |  |  |  | |
| 209 | 37 | 38 | 39 | 40 | 41 | 42 | 43 | 44 | 45 |  |  |  |  |  |  |  |  | |
| 217 | 46 | 47 | 48 | 49 | 50 | 51 | 52 | 53 | 54 | 55 |  |  |  |  |  |  |  | |
| 225 | 56 | 57 | 58 | 59 | 60 | 61 | 62 | 63 | 64 | 65 | 66 |  |  |  |  |  |  | |
| 233 | 67 | 68 | 69 | 70 | 71 | 72 | 73 | 74 | 75 | 76 | 77 | 78 |  |  |  |  |  | |
| 241 | 79 | 80 | 81 | 82 | 83 | 84 | 85 | 86 | 87 | 88 | 89 | 90 | 91 |  |  |  |  | |
| 249 | 92 | 93 | 94 | 95 | 96 | 97 | 98 | 99 | 100 | 101 | 102 | 103 | 104 | 105 |  |  |  | |
| 257 | 106 | 107 | 108 | 109 | 110 | 111 | 112 | 113 | 114 | 115 | 116 | 117 | 118 | 119 | 120 |  |  | |
| 269 | 121 | 122 | 123 | 124 | 125 | 126 | 127 | 128 | 129 | 130 | 131 | 132 | 133 | 134 | 135 | 136 |  | |
| 273 | 137 | 138 | 139 | 140 | 141 | 142 | 143 | 144 | 145 | 146 | 147 | 148 | 149 | 150 | 151 | 152 | 153 | |

Tab. S3 Start and end time of 105 time windows for summer-harvest crops in HHHR

| End  (DOY) | Start (DOY) | | | | | | | | | | | | | |
| --- | --- | --- | --- | --- | --- | --- | --- | --- | --- | --- | --- | --- | --- | --- |
|  | 65 | 73 | 81 | 89 | 97 | 105 | 113 | 121 | 129 | 137 | 145 | 153 | 161 | 169 |
| 65 | 1 |  |  |  |  |  |  |  |  |  |  |  |  |  |
| 73 | 2 | 15 |  |  |  |  |  |  |  |  |  |  |  |  |
| 81 | 3 | 16 | 28 |  |  |  |  |  |  |  |  |  |  |  |
| 89 | 4 | 17 | 29 | 40 |  |  |  |  |  |  |  |  |  |  |
| 97 | 5 | 18 | 30 | 41 | 51 |  |  |  |  |  |  |  |  |  |
| 105 | 6 | 19 | 31 | 42 | 52 | 61 |  |  |  |  |  |  |  |  |
| 113 | 7 | 20 | 32 | 43 | 53 | 62 | 70 |  |  |  |  |  |  |  |
| 121 | 8 | 21 | 33 | 44 | 54 | 63 | 71 | 78 |  |  |  |  |  |  |
| 129 | 9 | 22 | 34 | 45 | 55 | 64 | 72 | 79 | 85 |  |  |  |  |  |
| 137 | 10 | 23 | 35 | 46 | 56 | 65 | 73 | 80 | 86 | 91 |  |  |  |  |
| 145 | 11 | 24 | 36 | 47 | 57 | 66 | 74 | 81 | 87 | 92 | 96 |  |  |  |
| 153 | 12 | 25 | 37 | 48 | 58 | 67 | 75 | 82 | 88 | 93 | 97 | 100 |  |  |
| 161 | 13 | 26 | 38 | 49 | 59 | 68 | 76 | 83 | 89 | 94 | 98 | 101 | 103 |  |
| 169 | 14 | 27 | 39 | 50 | 60 | 69 | 77 | 84 | 90 | 95 | 99 | 102 | 104 | 105 |

Tab. S4 Start and end time of 75 time windows for autumn-harvest crops in HHHR

| End  (DOY) | Start (DOY) | | | | | | | | | | |
| --- | --- | --- | --- | --- | --- | --- | --- | --- | --- | --- | --- |
|  | 185 | 193 | 201 | 209 | 217 | 225 | 233 | 241 | 249 | 257 |  |
| 185 | 1 |  |  |  |  |  |  |  |  |  |  |
| 193 | 2 | 11 |  |  |  |  |  |  |  |  |  |
| 201 | 3 | 12 | 20 |  |  |  |  |  |  |  |  |
| 209 | 4 | 13 | 21 | 28 |  |  |  |  |  |  |  |
| 217 | 5 | 14 | 22 | 29 | 35 |  |  |  |  |  |  |
| 225 | 6 | 15 | 23 | 30 | 36 | 41 |  |  |  |  |  |
| 233 | 7 | 16 | 24 | 31 | 37 | 42 | 46 |  |  |  |  |
| 241 | 8 | 17 | 25 | 32 | 38 | 43 | 47 | 50 |  |  |  |
| 249 | 9 | 18 | 26 | 33 | 39 | 44 | 48 | 51 | 53 |  |  |
| 257 | 10 | 19 | 27 | 34 | 40 | 45 | 49 | 52 | 54 | 55 |  |

Tab. S5 Start and end time of 78 time windows for summer-harvest crops in the YZRB

| End  (DOY) | Start (DOY) | | | | | | | | | | | |
| --- | --- | --- | --- | --- | --- | --- | --- | --- | --- | --- | --- | --- |
|  | 33 | 41 | 49 | 57 | 65 | 73 | 81 | 89 | 97 | 105 | 113 | 121 |
| 33 | 1 |  |  |  |  |  |  |  |  |  |  |  |
| 41 | 2 | 13 |  |  |  |  |  |  |  |  |  |  |
| 49 | 3 | 14 | 24 |  |  |  |  |  |  |  |  |  |
| 57 | 4 | 15 | 25 | 34 |  |  |  |  |  |  |  |  |
| 65 | 5 | 16 | 26 | 35 | 43 |  |  |  |  |  |  |  |
| 73 | 6 | 17 | 27 | 36 | 44 | 51 |  |  |  |  |  |  |
| 81 | 7 | 18 | 28 | 37 | 45 | 52 | 58 |  |  |  |  |  |
| 89 | 8 | 19 | 29 | 38 | 46 | 53 | 59 | 64 |  |  |  |  |
| 97 | 9 | 20 | 30 | 39 | 47 | 54 | 60 | 65 | 69 |  |  |  |
| 105 | 10 | 21 | 31 | 40 | 48 | 55 | 61 | 66 | 70 | 73 |  |  |
| 113 | 11 | 22 | 32 | 41 | 49 | 56 | 62 | 67 | 71 | 74 | 76 |  |
| 121 | 12 | 23 | 33 | 42 | 50 | 57 | 63 | 68 | 72 | 75 | 77 | 78 |

Tab. S6 Start and end time of 78 time windows for autumn-harvest crops in the YZRB

| End  (DOY) | Start (DOY) | | | | | | | | | | | |
| --- | --- | --- | --- | --- | --- | --- | --- | --- | --- | --- | --- | --- |
|  | 193 | 201 | 209 | 217 | 225 | 233 | 241 | 249 | 257 | 265 | 273 | 281 |
| 193 | 1 |  |  |  |  |  |  |  |  |  |  |  |
| 201 | 2 | 13 |  |  |  |  |  |  |  |  |  |  |
| 209 | 3 | 14 | 24 |  |  |  |  |  |  |  |  |  |
| 217 | 4 | 15 | 25 | 34 |  |  |  |  |  |  |  |  |
| 225 | 5 | 16 | 26 | 35 | 43 |  |  |  |  |  |  |  |
| 233 | 6 | 17 | 27 | 36 | 44 | 51 |  |  |  |  |  |  |
| 241 | 7 | 18 | 28 | 37 | 45 | 52 | 58 |  |  |  |  |  |
| 249 | 8 | 19 | 29 | 38 | 46 | 53 | 59 | 64 |  |  |  |  |
| 257 | 9 | 20 | 30 | 39 | 47 | 54 | 60 | 65 | 69 |  |  |  |
| 265 | 10 | 21 | 31 | 40 | 48 | 55 | 61 | 66 | 70 | 73 |  |  |
| 273 | 11 | 22 | 32 | 41 | 49 | 56 | 62 | 67 | 71 | 74 | 76 |  |
| 281 | 12 | 23 | 33 | 42 | 50 | 57 | 63 | 68 | 72 | 75 | 77 | 78 |

Tab. S7 Start and end time of 21 time windows for early rice in the YZRB

| End (DOY) | Start(DOY) | | | | | |
| --- | --- | --- | --- | --- | --- | --- |
|  | 153 | 161 | 169 | 177 | 185 | 193 |
| 153 | 1 |  |  |  |  |  |
| 161 | 2 | 7 |  |  |  |  |
| 169 | 3 | 8 | 12 |  |  |  |
| 177 | 4 | 9 | 13 | 16 |  |  |
| 185 | 5 | 10 | 14 | 17 | 19 |  |
| 193 | 6 | 11 | 15 | 18 | 20 | 21 |

Tab. S8 The optimal thresholds of each province in in the NEC-IMR, the HHHR and the YZRB.

| Thresholds  (%) | Autumn-harvest crops | | | Summer-harvest crops | | | Early rice | | |
| --- | --- | --- | --- | --- | --- | --- | --- | --- | --- |
|  | Drought-covered area | Drought-damaged area | Crop failure area | Drought-covered area | Drought-damaged area | Crop failure area | Drought-covered area | Drought-damaged area | Crop failure area |
| Heilongjiang | 70% | 61% | 44% |  |  |  |  |  |  |
| Jilin | 69% | 62% | 48% |  |  |  |  |  |  |
| Liaoning | 70% | 62% | 52% |  |  |  |  |  |  |
| Inner Mongolia | 59% | 50% | 40% |  |  |  |  |  |  |
| Shandong | 45% | 41% | 28% | 56% | 51% | 34% |  |  |  |
| Hebei | 45% | 41% | 28% | 56% | 51% | 34% |  |  |  |
| Jiangsu | 41% | 39% | 23% | 45% | 36% | 19% |  |  |  |
| Henan | 53% | 42% | 31% | 41% | 34% | 23% |  |  |  |
| Anhui | 29% | 19% | 10% | 47% | 38% | 30% |  |  |  |
| Sichuan | 60% | 55% | 42% | 83% | 71% | 42% |  |  |  |
| Chongqing | 49% | 42% | 34% | 76% | 67% | 50% |  |  |  |
| Hubei | 42% | 36% | 21% | 76% | 60% | 24% |  |  |  |
| Hunan | 36% | 25% | 18% | 83% | 81% | 73% | 81% | 69% | 16% |
| Jiangxi | 25% | 16% | 10% | 90% | 85% | 50% | 82% | 51% | 19% |
